# Model Studies on Load-Settlement Characteristics of Coarse-Grained Soil Treated with Geofiber and Cement

**DOI:** 10.3390/polym10060621

**Published:** 2018-06-05

**Authors:** Yan Li, Lei Su, Xianzhang Ling, Jiahui Wang, Yingzi Yang

**Affiliations:** 1 School of Civil Engineering, Harbin Institute of Technology, Harbin 150090, China; liyancs@126.com (Y.L.); wangjiahui11@126.com (J.W.); yzyang@hit.edu.cn (Y.Y.); 2 School of Civil Engineering, Qingdao University of Technology, Qingdao 266033, China; sl_hit@163.com

**Keywords:** fiber reinforcement, fiber-cement reinforcement, laboratory subgrade model test, load-settlement characteristics

## Abstract

This study aims to verify the effectiveness of fiber reinforcing with and without cement on settlement controlling of subgrade models, and to investigate the effect of fiber reinforcement on the load-settlement behavior of subgrade models. To this end, laboratory subgrade model tests were conducted under different static vertical loads. Three subgrade models composed of different fillers were constructed in a rigid concrete tank, and the internal earth pressures and settlements at different depths were measured through an earth pressure cell and settlement plate. Results show that the fiber-reinforced model keeps a slight difference to the unreinforced model in terms of earth pressure distribution under lower applied surface pressure. However, the earth pressure at various locations under each surface pressure was obviously lower than that of the other two models due to the combined effect of fiber and cement. In addition, for the unreinforced subgrade model, the 60 cm settlement domain was restricted within 40 cm depth through fiber-cement and fiber reinforcing, and the total settlement under 100 kPa was decreased by 48.5% and 30.8%, respectively. Moreover, reinforced models present with different settlement deformation features. The inflection points, after which the rate of settlement decreased with increasing applied surface pressure, were observed in the pressure-settlement curves. Under 200 kPa, the fiber-cement and fiber reinforcement decreased the total settlement of the unreinforced model by 61.4% and 34.7%, respectively. The greater applied surface pressure, the more efficient was fiber-cement reinforcing in settlement controlling.

## 1. Introduction

The general trend of the world’s railway development is toward higher speed and heavier load among traffic transportation. As the most basic and main component of railway track systems, the subgrade supporting the upper track structure (i.e., ballast bed, sleepers, tracks and train loads) plays an important role in keeping good performance of the railway track under repeated traffic loads, and faces more strict requirements during the whole construction. The track evenness induced by the subgrade settlement directly is a widespread problem in geotechnical engineering and always brings trouble for railway transportation. Thus, how to efficiently control and minimize the subgrade settlement has become a common concern in railway construction.

In geotechnical engineering, the most common technique to improve soil engineering performance is mixing fine-grained and granular soils with cement or gypsum in a suitable proportion. In general, cemented soils show a good compressive strength but a brittle behavior and poor tensile strength. Under cyclic loading or lateral earth pressure, the stabilized material may suffer to a brittle or sudden failure without any plastic deformation. Such brittle failure patterns and poor tensile behavior can be prevented by adding the short fibers into cemented soils [1,2]. In the aspect of experimental study on fiber reinforcement, investigators have conducted a great number of unconfined compression tests, triaxial tests, California bearing ratio tests, direct shear tests and tensile tests on fiber-reinforced soil in the last few decades [3,4,5,6,7,8,9,10,11,12,13,14,15]. All these previous investigations indicate that fiber reinforcement can be considered as a good soil improvement technique, and such soil presents great mechanical performance. Meanwhile, as the added fibers increase, the compressive and tensile strength, ductility and toughness of soil specimens will be enhanced [16,17,18,19]. Experimental results indicated that fibers can be easily added and mixed with soil and provide an isotropic increase in shear/tension strength of compound soil without introducing potential slide surfaces. Several researchers [20,21,22,23] studied the combined effect of fibers with other admixtures such as cement, lime and fly ash on granular or fine-grained soils, and the experimental results demonstrated that such a combined effect provided much better performance (i.e., increased soil strength), compared with the reinforcement of fibers and additives separately. In the aspect of theoretical research, Tang et al. [8] analyzed the micromechanism of interfacial interaction between cemented soil particles and fibers through scanning electron microscopy and presented a theoretical model, which helps to interpret the tensile strength mechanism of improved cemented soil with fiber. Foraboschi [24,25] presented a multiscale analytical model, which described the delayed debonding considering the interaction of bond shear stress and time to predict the lifetime of concrete members externally reinforced with fiber-reinforced polymers (FRP). The theoretical finding suggests that the reinforcement will lose the bond (delayed debonding) due to the microcracks within cover concrete developed up to a critical crack length.

However, the previous laboratory tests on fiber-reinforced soils were mainly carried out with small-sized cylindrical (50 mm in diameter and 100 mm high) or rectangular specimens (40 mm × 40 mm × 160 mm). Furthermore, very limited information has been reported related to the load-settlement behavior of fiber-reinforced soils. Santoni and Webster [26] conducted the field tests on sand-fiber layers with different thicknesses under the C-130 aircraft wheel loads and found that the fibers can significantly increase the pavement resistance to rutting. Consoli et al. [27,28] performed the plate load tests on fiber-reinforced sand layers and concluded that the addition of fibers considerably improved the soil load-settlement behavior by spreading the stresses to a broader area. According to the field tests mentioned above, very few studies have been reported about the effect of fibers combined with cement on load-settlement behavior of stabilized soils. Especially, the characteristics of stress distribution and load-settlement at different depths inside the subgrade reinforced by fibers with and without cement have not been reported.

The main objectives of this study are to explore the effectiveness of fiber reinforcing with and without cement on settlement controlling of subgrade models, and to investigate the effect of fiber reinforcement on the load-settlement behavior of subgrade models. To this goal, the laboratory subgrade model tests for coarse-grained soils reinforced by polypropylene fibers with and without cement have been performed under different static vertical loads. The earth pressures and settlements were measured at different depths along the vertical central line of subgrade model and the experimental results were compared and discussed.

## 2. Materials and Experimental Procedure

### 2.1. Coarse-Grained Soil

**Figure 1:**
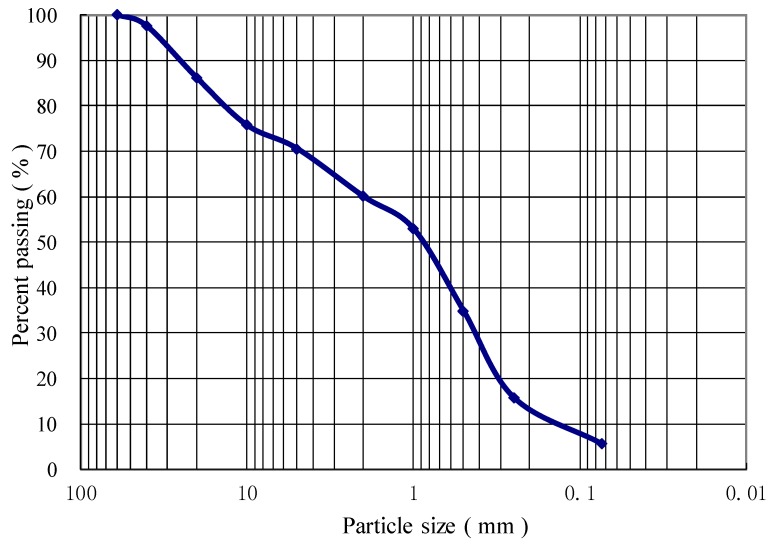
Particle size distribution curve.

The soil employed in this study was coarse-grained soil from a borrow pit of railway construction, which was used as filling material for the subgrade of Shenhua Heavy Haul Railway of Ordos section located in Inner Mongolia Autonomous Region of China. The soil has a coefficient of curvature (Cc) of 0.54, coefficient of uniformity (Cu) of 11, effective grain size (D10) of 0.18 mm and control grain size (D60) of 1.99 mm. The optimum water content of soil is determined at 8.2% and its maximum dry density is 2.14 g/cm^3^ based on the results of compaction test. Such coarse-grained soil is classified as poorly graded gravel sand according to the Code for Design on Subgrade of Railway of China (TB10001-2005/J447-2005), and its grain-size distribution curve is shown in Figure 1.

### 2.2. Polypropylene Fiber and Cement

The monofilament polypropylene fiber was used in the whole experimental study and some geometrical and physical properties of this fiber provided by the manufacturer are listed in Table 1. The fiber content (relative to weight of soil) used herein was 0.3%.

The cement adopted in the test was ordinary Portland cement and its physical properties and chemical compositions are given in Table 2. The cement content (relative to weight of soil) used in this test was 7%.

### 2.3. Model Test Description

**Figure 2:**
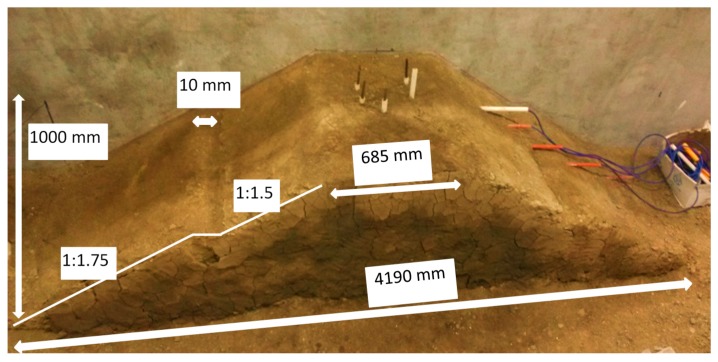
Profile photo of subgrade model (unit: mm).

In total, three subgrade model tests were conducted in a rigid concrete tank with a 5000 mm length, 2500 mm width and 3000 mm height. All three subgrade models were constructed with the same geometric dimension, that is, 685 mm length in the subgrade top (4190 mm length in the subgrade bottom), 1000 mm width, 1000 mm height. The unreinforced coarse-grained soil, fiber-reinforced soil and fiber-cement-reinforced soil were selected as the experimental materials to construct different subgrade models. The profile photo of the subgrade model is shown in Figure 2.

**Figure 3:**
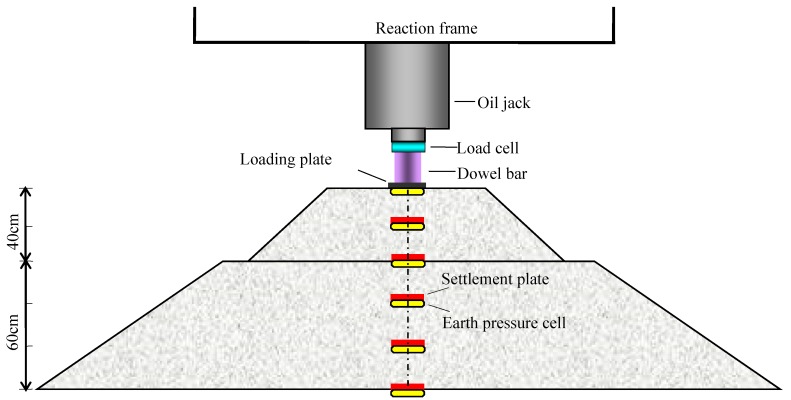
Loading and measuring system of the subgrade model.

The test load was applied by an oil jack supported against a rigid reaction frame and measured using a calibrated load cell. A square rigid loading plate with dimensions of 150 mm × 150 mm, and 25 mm thickness, was placed at the center of the top surface of the subgrade model and used for transferring the external load to the model. The vertical displacement of loading plate (i.e., the total settlement of subgrade model) was directly measured by a dial gauge. The internal settlement of predetermined depth of the subgrade model was measured by five settlement plates, which were composed of steel base plates with the dimensions of 100 mm length × 100 mm width × 10 mm thickness, measuring iron rods with diameter of 5 mm and plastic sleeves with diameter of 10 mm. The vertical displacement of settlement plates was measured through five dial gauges placed on the top surface of iron rods. The internal pressure of the subgrade model was measured through five earth pressure cells (Kingmach measurement & monitoring technique co., ltd, Changsha, China) located at the different designated depths along the vertical central line of the model. The loading and measuring system was shown in Figure 3.

The load was applied in equal increments of 10 and 20 kPa, which were one tenth of the two designed loadings of 100 and 200 kPa. After each load increment, the resulting settlements and variation of earth pressure cells were recorded at a fixed time interval of 60 min. Each increment was maintained until the settlement difference in 60 min was less than 0.05 mm. The loading period of the model test was stopped as the designed loads were reached.

### 2.4. Model Construction

The soil material was dried and prepared in a rotating drum mixer (Midfielder lifting equipment co., ltd, Baoding, China), by firstly mixing the dry coarse-grained soil, polypropylene fiber and cement to achieve a uniform mixture. Then, the optimum moisture content was achieved by adding the water to the mixture to get a homogenous fiber-cement improved soil. In the process of preparation, the unreinforced soil and fiber-reinforced soil were obtained through mixing with no fiber and cement, or cement. Before the construction of the subgrade model, a 500-mm-thick coarse-grained soil layer was built in two consecutive layers, each 250 mm thickness, by using a small-sized hand-guided vibratory roller (Fulu tong machinery equipment co., ltd, Jining, China) to reach a relative density of 90%. Above this layer, the subgrade model was constructed in five consecutive steps of 200 mm each, with each layer being compacted to a relative density of 95%. During the construction of the model, the settlement plates and earth pressure cells were embedded at the predetermined depth located at the soil interface of the subgrade model. After completing the model construction, the subgrade was allowed to cure for two weeks before being tested, and measures of water spraying and covering were taken to make sure that the model maintained the proper water content.

## 3. Results and Discussions

### 3.1. Pressure Distribution Characteristics of Subgrade Models

**Figure 4:**
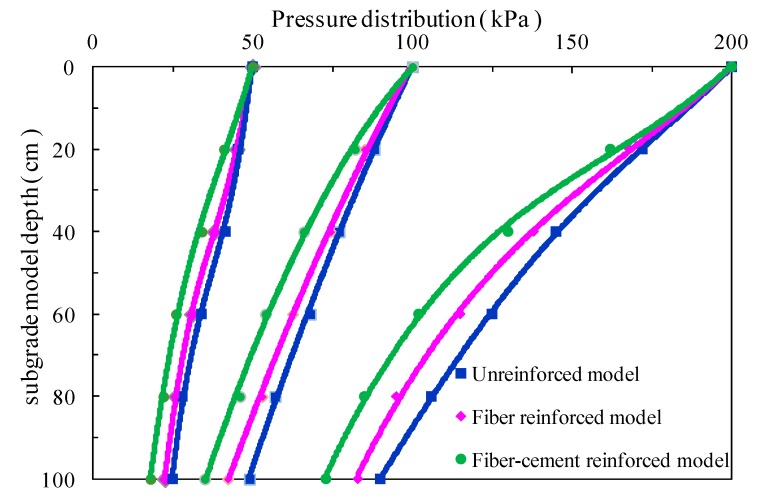
Relationship curves between pressure distribution and subgrade model depth.

Figure 4 presents the earth pressure distribution along the depth under different static surface pressures. A similar trend that the earth pressure decreased nonlinearly with increasing of depth was observed in all subgrade models. For lower surface pressure of 50 kPa, there is only a slight difference between the pressure distribution curves of the unreinforced model and fiber-reinforced model for the earth pressures measured in the same locations at different depths. The combined action of cement and fibers made the earth pressure measured at each checkpoint lower than that of unreinforced and fiber-reinforced models, thus resulting in the pressure distribution curve of the fiber-cement-reinforced model relatively differing from curves of the other two. From Figure 4, the earth pressure difference for unreinforced and reinforced-models increased with increasing applied surface pressure. As the applied surface pressure rises, the pressure distribution curves are smoother. For higher surface pressure of 200 kPa, at 100 cm depth, the earth pressures of unreinforced, fiber-reinforced and fiber-cement-reinforced subgrade models were 90.1, 80.2 and 73.2 kPa, respectively. Using improvement measure of fiber and fiber-cement models, the earth pressure decreased by approximately 11% and 18.6%, in comparison with that of the unreinforced model. For the lower surface pressure of 50 kPa, the fiber reinforcement with and without cement reduced the earth pressure by 12.7% and 6.3% at 100 cm depth, respectively, in comparison to the unreinforced model. It indicates that the greater the applied surface pressure, the better the load diffusion capacity of fiber reinforcing. Fibers play an active role in transferring and extending the static load on the top surface of subgrade models, and the benefit of fiber reinforcing is found to be more pronounced by the addition of cement.

The reason for phenomena mentioned above might be that the relative slippage between fibers and soil particles is very limited owing to the low compaction of soil particles under the lower surface pressure. During this stage, the fibers could not interact well with the soil, therefore, the effect of fiber reinforcement is not obvious. For the fiber-cement-reinforced model, the addition of cement increases the stiffness of the subgrade model which redistributes the applied surface pressure over a larger zone. Under higher applied surface pressure, the subgrade model is more compacted. As the fibers are elongated, the interfacial friction and bonding force between the soil particles and fibers restrains the relative movement of soil particles and thus helps to disperse the concentrated vertical loads over a greater zone. The actual pressure transferred to a certain soil layer decreases. For the fiber-cement-reinforced model, the elongated fibers further strengthen the characteristic of load diffusion.

### 3.2. Settlement Characteristics of Subgrade Models

#### 3.2.1. Settlement under Surface Pressure of 100 kPa

**Figure 5:**
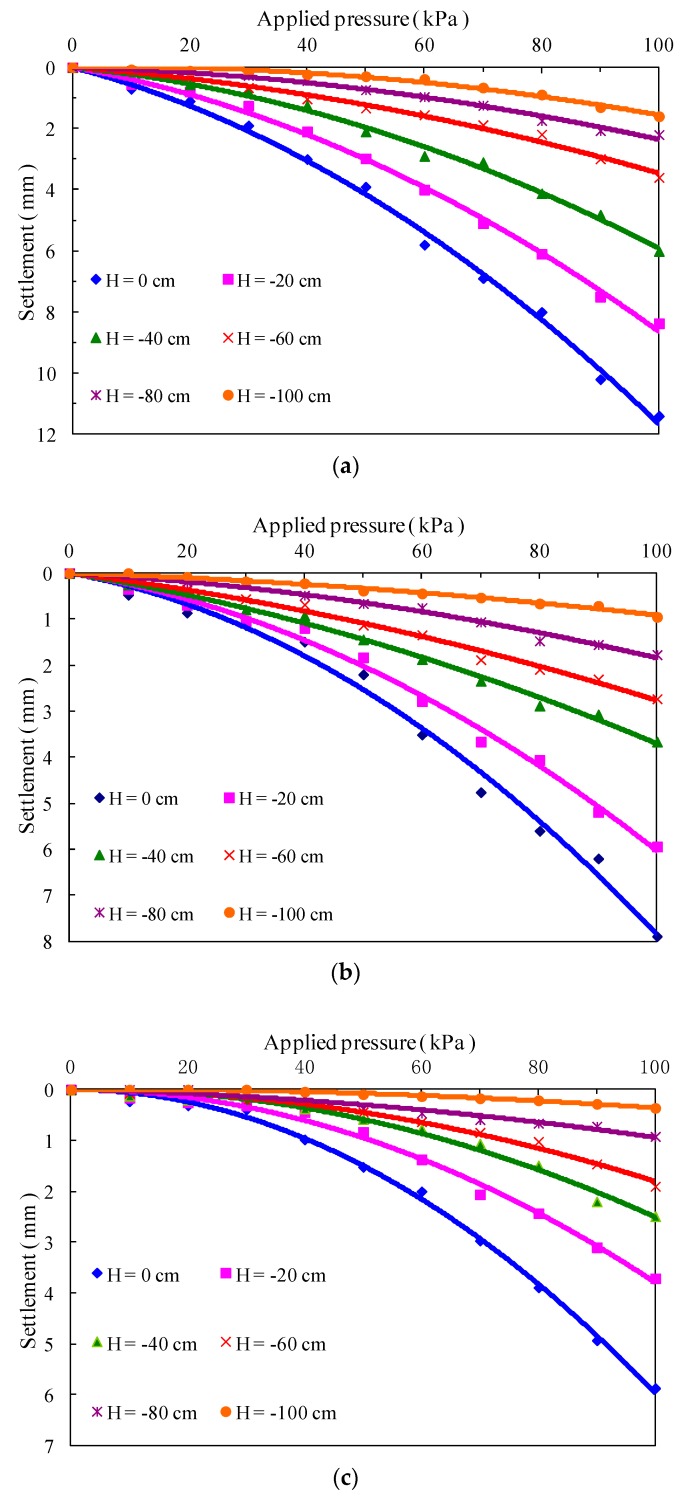
Applied pressure-settlement curves for unreinforced and reinforced subgrade models. (**a**) Unreinforced subgrade model; (**b**) fiber-reinforced subgrade model; (**c**) fiber-cement-reinforced subgrade model.

**Figure 6:**
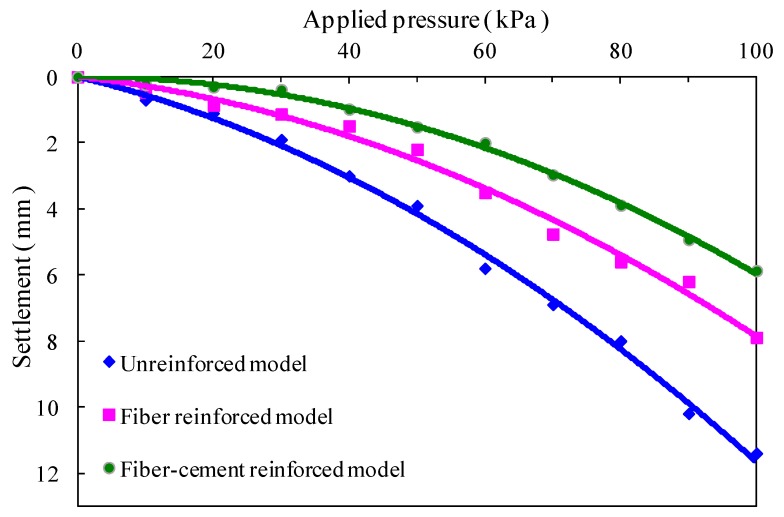
Comparison of applied surface pressure-settlement curves of the top surface for three subgrade models.

Figure 5 presents the pressure-settlement curves for soil stratum at different depths of three subgrade models. Overall, three subgrade models exhibit basically the same pressure-settlement features, which demonstrates that the settlement of soil stratum at different depths increases nonlinearly with the increase of applied pressure, and the settlement deformation reaches the maximum near the model surface. In addition, the main settlement of the unreinforced subgrade model was observed to occur in the range of 60 cm depth, but it is restricted within a depth of 40 cm by fiber reinforcement with and without cement. For the fiber-reinforced model, the pressure-settlement curves were relatively gentle as compared with that of the unreinforced model, and all the settlement of each soil stratum at the same depth is lower than that of the unreinforced model. Moreover, a similar pattern is found for the fiber-cement-reinforced subgrade model, which presents smaller settlement deformation. As shown in Figure 6, under the applied surface pressure of 100 kPa, the total settlements of the top surfaces of the three subgrade models separately were 11.4, 7.9 and 5.9 mm. It can be deduced that the fiber reinforcement with and without cement decreases the settlement of the unreinforced model by 48.5% and 30.8%, respectively.

The contribution of fiber reinforcement in reducing the settlement of soil stratum of the subgrade model is mainly due to the interlocking of fibers with soil particles, which resist the relative movement between particles by interfacial friction and bonding force. However, unlike the combined reinforcement effect of fibers and cement, the fiber reinforcing does not change the soil microstructure and fill the soil void. [8] studied the interfacial interactions between fibers and soil particles using scanning electron microscopy, and the experimental results confirmed that the hydration products of cement can fill the soil void and bind the soil particles with fibers together. Therefore, by adding the cement, a denser soil matrix and better interaction of fibers with soil particles are formed, which results in a stronger interfacial friction and bonding force between fibers and soil particles. Such a strengthening effect further improves the pressure distribution on the subgrade model and reduces the settlement.

#### 3.2.2. Settlement under Applied Surface Pressure of 200 kPa

**Figure 7:**
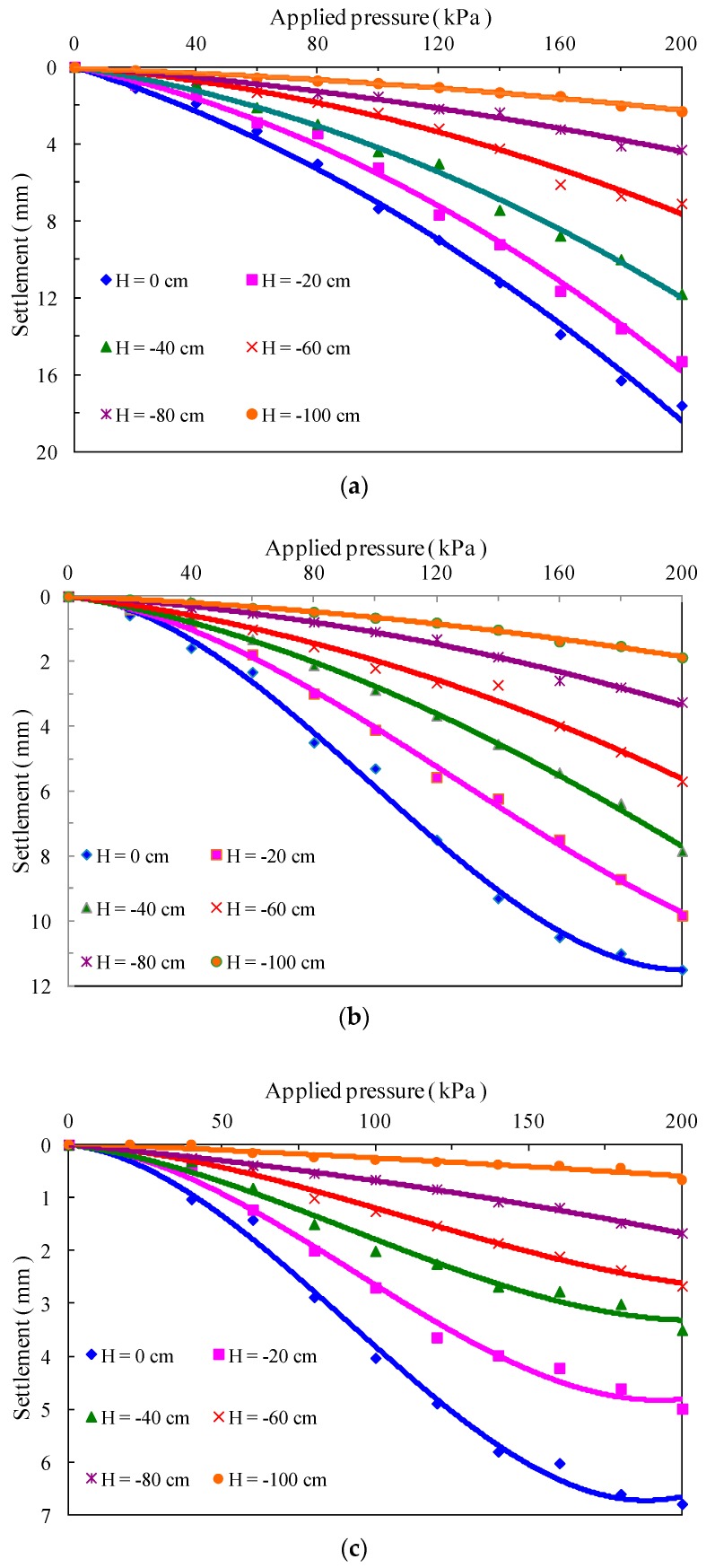
Applied pressure-settlement curves for unreinforced and reinforced subgrade models. (**a**) Unreinforced subgrade model; (**b**) fiber-reinforced subgrade model; (**c**) fiber-cement-reinforced subgrade model.

Figure 7 shows the pressure-settlement curves for unreinforced and reinforced subgrade models under the applied surface pressure of 200 kPa. By comparison of Figure 5a and Figure 7a, it is observed that two unreinforced subgrade models have the same deformation features. The curve shape of pressure-settlement does not change noticeably, although the settlement of soil stratum at different depths increased significantly as the applied surface pressure increased from 100 to 200 kPa. For the fiber-reinforced model, as the applied surface pressure continually increases, an inflection point corresponding to the pressure of 140 kPa is observed in the pressure-settlement curve on the top surface of the subgrade model, after which the rate of settlement decreases with increase of applied surface pressure. The inflection points and behavior of decreasing settlement rate are also found in the curves of soil stratum within a depth of 40 cm for the fiber-cement-reinforced model. Moreover, at the same depth of subgrade models, the maximum settlement occurs in the unreinforced soil stratum, and settlement of the fiber-cement-reinforced soil stratum is relatively small. The shallow soil layer will lead to the greater settlement differences among three subgrade models.

**Figure 8:**
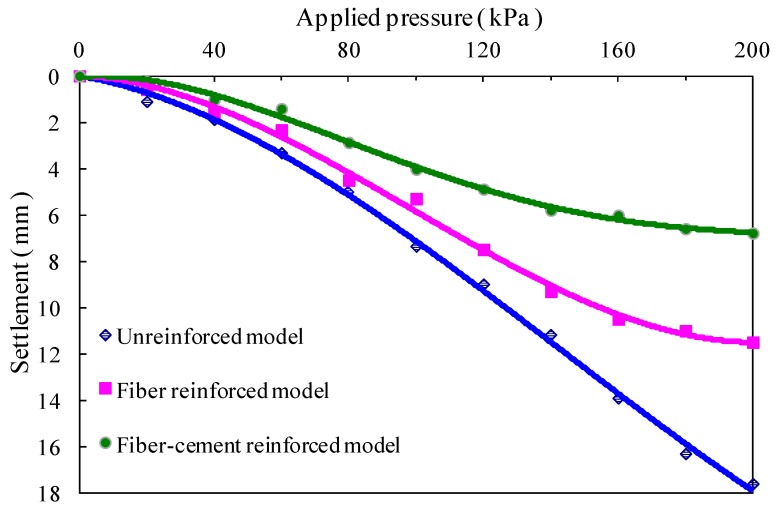
Comparison of applied surface pressure-settlement curves of the top surface for three subgrade models.

The comparison of pressure-settlement curves of the top surface for three subgrade models under the applied surface pressure of 200 kPa is shown in Figure 8. It could be seen that, in the range of 100 to 200 kPa, the pressure-settlement response of the unreinforced model is almost linear, while the response of the reinforced models demonstrates the nonlinear change due to the reduction of settlement rate. With the provision of fiber reinforcement, there is a substantial reduction in the top surface settlement of the unreinforced subgrade model, and its settlement was decreased by 34.7%, from 17.6 to 11.5 mm. Furthermore, the added cement dramatically enhances the ability of fiber reinforcing in terms of settlement inhibition. The total settlement of the top surface of the unreinforced model reduces by 61.4%, from 17.6 to 6.79 mm, through fiber-cement reinforcing.

From the experimental results presented in Figure 6 and Figure 8, it is of interest to note that the beneficial effect of fiber reinforcement with and without cement is evident, especially at larger settlement of subgrade models. The fiber reinforcement decreases the total settlement of the unreinforced model by 30.8%, and increases to 34.7% as the applied surface pressure increased from 100 to 200 kPa. Likewise, using the fiber-cement reinforcement, the reduction of settlement increased from 48.5% to 61.4%, which exhibited a potential ability of keeping the subgrade able to maintain a small settlement deformation under high applied surface pressure.

For the unreinforced subgrade model, the settlement increased greatly with increasing applied surface pressure, which is mainly due to the frictional and bonding force of soil matrix being unable to restrict the rearrangement of particles induced by growing surface pressures, although the prior compaction under pressure of 100 kPa reduces the soil void and produces a relatively denser subgrade. For the fiber-reinforced model, the role of fibers is decided by the performance of the soil matrix. As the soil particles are compacted denser with increasing pressure, the interfacial contact area between fibers and particles also increases, which gives rise to an improvement of interfacial interaction. Consequently, the function of fibers in decreasing relative movement of soil particles can spread the vertical pressure and mitigate the subgrade settlement. However, as the external load was performed on the top surface of the subgrade model, the internal microcracks were also formed and continually grew with increasing of the external load. Part of the fibers will be pulled out from soil particles and lose the bond (delayed debonding), while a critical crack length was reached [24,25]. Thus, the effect of weakening fiber reinforcement causes a further settlement of the fiber-reinforced model. The presence of cement inclusion produces a stronger frictional interaction between soil particles and fibers, which efficiently impedes the sliding of fibers from the soil matrix and increases the effect of reinforcement due to some of the fiber surface and soil particles being wrapped around by cement hydration products. Furthermore, the products of cement hydration occupy part of the soil void, and as the model subgrade is compressed more densely, the space for the relative movement of soil particles is less and less. As a result, more and more compaction energy is needed for overcoming the interfacial resistance of fiber-cement-soil composites. It is clear to see from the experimental results that, for the fiber-cement-reinforced subgrade model, the compaction energy of 200 kPa is insufficient to cause great movement of soil particles, which leads to the smallest settlement compared with the other two subgrade models.

## 4. Conclusions

In this study, the laboratory subgrade model tests under different static vertical loads were conducted to investigate the reinforcing effect of fibers with and without cement on the load-settlement behavior of soils. The main experimental results can be summarized as follows:

(1) All three subgrade models possess a similar distribution trend of the earth pressure, which decreased nonlinearly with increasing depth. The measured earth pressure of the fiber-reinforced model at different depths showed only a slight difference, compared with the subgrade model without reinforcing under lower surface pressure. However, the earth pressure at each location under surface pressure was obviously lower than that in the other two models due to the combined effect of fiber and cement. The greater surface pressure produced the better load diffusion capacity for fiber–cement reinforcing.

(2) Under the applied surface pressure of 100 kPa, the main settlement domain of the unreinforced subgrade model was observed to occur in the range of 60 cm depth, and this was restricted within 40 cm depth by fiber reinforcement with and without cement. In addition, the settlement of each soil stratum of the reinforced model was lower than that of the unreinforced model, and the total settlement was decreased by 48.5% and 30.8% through fiber-cement and fiber reinforcing, respectively, compared with the unreinforced model.

(3) Reinforced models present a different settlement deformation feature, compared with the unreinforced model. The inflection points, after which the rate of settlement decreased with increase of applied surface pressure, were observed in the pressure-settlement curves corresponding to 140 kPa. The fiber-cement reinforcement behaves better than fiber reinforcing in terms of settlement inhibition. The total settlement separately decreased by 61.4% and 34.7%, compared with the unreinforced model. The greater applied surface pressure, the more efficient was fiber-cement reinforcing in settlement controlling.

(4) Fibers play an active role in dispersing external loadings and controlling subgrade settlement, and the benefit of fiber reinforcing is found to be more pronounced by the addition of cement.

## Figures and Tables

**Table 1:** Performance parameters of polypropylene fiber.

Parameter	Value
Fiber type	Single fiber
Specific gravity	0.91
Diameter (mm)	0.031
Length (mm)	7
Breaking tensile strength (MPa)	330–370
Elongation at break (%)	30
Modulus of elasticity (MPa)	3500
Fusion point (°C)	165
Burning point (°C)	590

**Table 2:** Physical properties and chemical compositions of cement.

Compressive Strength, 28-day	CaO	SiO_2_	Al_2_O_3_	Fe_2_O_3_	MgO	SO_3_
32.5 MPa	63.6%	20.6%	3.2%	4.5%	2.7%	2.9%
